# Healthy elderly Singaporeans show no age-related humoral hyporesponsiveness nor diminished plasmablast generation in response to influenza vaccine

**DOI:** 10.1186/s12979-018-0137-4

**Published:** 2018-11-12

**Authors:** Xavier Camous, Lucian Visan, Crystal Tan Tze Ying, Brian Abel, Ma Shwe Zin Nyunt, Vipin Narang, Michael Poidinger, Christophe Carre, Sanie Sesay, Nabil Bosco, Nicolas Burdin, Paul Anantharajah Tambyah, Ng Tze Pin, Anis Larbi

**Affiliations:** 1 Biology of Aging Laboratory, Singapore Immunology Network, Agency for Science Technology and Research, Singapore, Singapore; 2 Sanofi Pasteur, Research and Non-Clinical safety, Marcy L’Etoile, France; 3 Immunomonitoring Platform, Singapore Immunology Network, Agency for Science Technology and Research, 8A Biomedical Grove, Singapore, 138648 Singapore; 4 Gerontology Research Programme, Department of Psychological Medicine, National University of Singapore, Singapore, Singapore; 5 Sanofi Pasteur, Clinical Sciences, Marcy L’Etoile, France; 6 Nestle Research Center Asia, 21 Biopolis Road, Singapore, Singapore; 7 Department of Infectious Diseases, National University Health System, Singapore, Singapore

**Keywords:** Aging, Influenza vaccination, Stratification, Co-morbidity

## Abstract

**Abstract:**

Improving influenza vaccine efficacy is a priority to reduce the burden of influenza-associated morbidity and mortality. By careful selection of individuals based on health we show sustained response to influenza vaccination in older adults. Sustaining health in aging could be an important player in maintaining immune responses to influenza vaccination.

**Trial registration:**

NCT03266237. Registered 30 August 2017, https://clinicaltrials.gov/ct2/show/NCT03266237.

## Influenza vaccination responses in older adults

Influenza infection is associated with considerable morbidity annually and the elderly are amongst those at highest risk of serious outcomes [1–3]. Annual influenza vaccination is a strategy endorsed by the World Health Organization [4], and is most effective when the vaccine strains closely match the circulating influenza viruses [5]. However, the antibody response and protection elicited by the vaccine in the elderly is modest at best [6], though recent studies suggest that increasing the dose of antigen in subunit vaccines significantly improves efficacy [7]. Several factors have been proposed to explain hyporesponsiveness to influenza vaccination in the elderly, including host-related factors like genetics, immune status, health status, frailty and nutritional status [8]. The complex changes in the immune system occurring with age collectively termed immunosenescence, affects innate and adaptive immunity and may contribute to the decreased efficacy of vaccines in the elderly [9, 10]. Recent data also suggest that age-associated decline in antibody responses could reflect the effect of repeated annual influenza vaccination rather than age or frailty [11], however this hypothesis is being queried [12]. Systems biology approaches have been utilized in recent studies to acquire a global picture of vaccine-induced immunity in humans, which has enabled the identification of early innate signatures predicting vaccine immunogenicity and the elucidation of novel mechanisms of immune regulation [13]. Several reports have established age-dependent predictive signatures of influenza vaccine responses, however such studies typically considered the elderly as a homogeneous population, which is clearly not the case [14]. We address this concern in the current study and show data confirming that a healthy elderly population, selected for lack of comorbidity and not just chronology, is likely to respond to influenza vaccination.

## Description of the study

The SENIEUR protocol clearly suggested that addressing chronological ageing alone was insufficient in immunogerontological studies [15] as the elderly of the SENIEUR category (healthy elderly as defined by the SENIEUR protocol) displayed similar immune capacity than younger counterparts (in vitro). We took advantage of the Singapore Longitudinal Ageing Study (SLAS), which is an ongoing population-based cohort study of aging and health among Chinese adults aged 55 and above, to select presumed healthy elderly subjects. The SLAS design and description of the cohort demographics and health profile have been described previously [16]. Individuals with no signs of frailty were selected using Fried’s criteria, which assesses five dimensions hypothesized to reflect systems whose impaired regulation underlies frailty, namely unintentional weight loss, exhaustion, muscle weakness, slowness while walking, and low levels of activity (i.e. Fried’s score of 0 to 5) [17]. Additionally, we excluded individuals with a history of dementia, cancer or cardiovascular disease [18], as well as those with any recent infection or any flu vaccine administration for at least 6 months before the study (Table 1). The healthy elderly individuals (*n* = 22) were aged between 65 and 84 years (mean = 72.4 years) and the young group (*n* = 29) was represented by individuals of the same ethnicity (Chinese) aged between 23 and 33 years (mean = 29.1 years). The healthy elderly group showed a level of activity similar to the young group as measured by actigraphy, while significant differences were observed for biomarkers such as cytomegalovirus (CMV) seropositivity (100% in older adults and 45% in the young group, *p* < 0.0001), the CD4/CD8 ratio (*p* < 0.01) and the level of C-Reactive Protein (CRP, p < 0.01) (Table 1).

**Table 1:** Study subject characteristics

	Young cohort (*N* = 29)	Healthy elderly cohort (*N* = 22)	*p*
Demography			
Age	27.97 (23–33)	72.41 (65–84)	****
Gender	18 females (63.3%)	10 females (45.5%)	*ns*
BMI	22.8 (16.2–29.1)	23.6 (17.4–32.6)	*ns*
Physical activity (% of time)			
Sedentary	60.3 (46.5–98.9)	60.2 (43.4–92.5)	*ns*
Light activity	35.1 (0.9–47.9)	35.2 (7.4–48.4)	*ns*
Moderate activity	4.5 (0.1–11.1)	3.8 (0.1–15.4)	*ns*
Vigorous activity	0.1 (0–1.8)	0.8 (0–12.1)	*ns*
Clinical			
CMV positivity	13 (45%)	22 (100%)	****
CD4/CD8 ratio	1.33 (0.62–2.42)	2.94 (0.52–14.71)	**
CRP (mg/L)	1.2 (0.2–4.1)	2.7 (0.3–9.3)	**
Comorbidities	–	0.36 (0–1)	*–*
MMSE/MoCA	–	28 (22–30) / 25.59 (18–30)	*–*
Pre/Post FEV1/FVC	–	0.73 (0.5–0.91)/0.71 (0.29–0.85)	*–*
Medications	–	1.05 (0–4)	*–*
Hospitalizations	–	0	*–*
MNA	–	12.8 (11–14)	*–*

*BMI* body mass index, *CMV* human cytomegalovirus, *CRP* C-reactive protein, *MMSE* mini mental state examination, *MoCA* Montreal cognitive assessment, *FEV* forced expiratory volume, *FVC* forced vital capacity, pre/post inhalation of a bronchodilator, *MNA* mini nutritional assessment score. Physical activity was measured during 2 weeks using actigraphy watches (Phillips Respironics). Comorbidities are expressed as the number of diagnosed conditions, medications are defined as the number of prescribed drugs and hospitalizations is the number of time the individual have been hospitalized in the past year

## Sustained antibody titers following influenza vaccination of healthy elderly

**Fig. 1:**
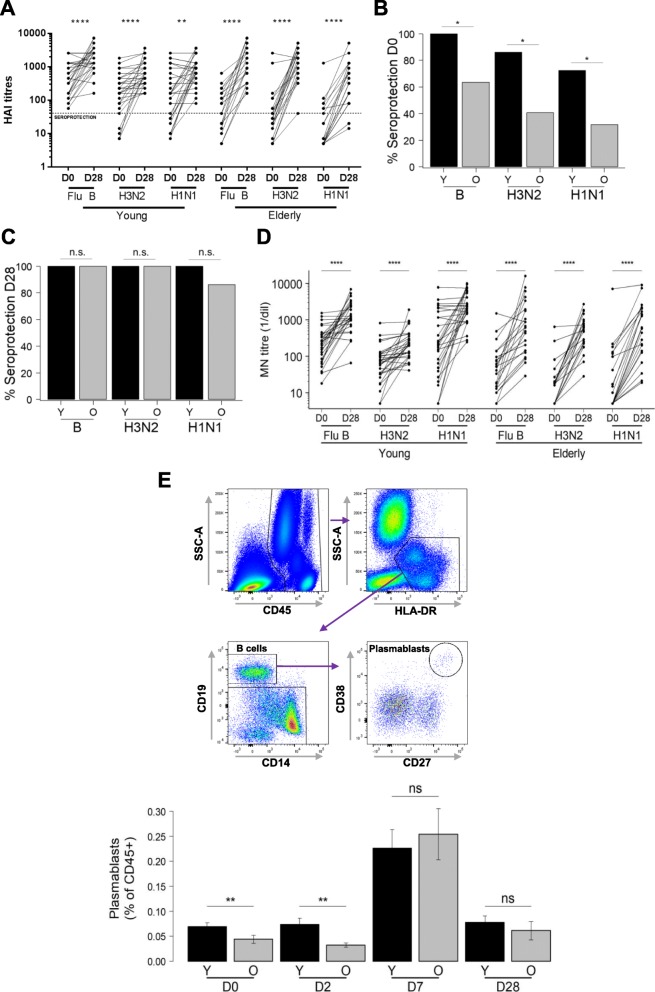
Humoral immunity to influenza vaccination. **a** Graph showing the basal (D0) HAI titers and the response at day 28 (D28) in young (left) and old (right) vaccinees for each one of the influenza virus strains after vaccination; paired t-test applied. Seroprotection of subjects indicated by the dotted line for HAI titers ≥40. **b**, **c** % Seroprotection in young (Y) and healthy elderly adult (O) vaccinees at baseline (D0) and day 28 (D28) for each one of the three influenza virus strains; significances were calculated using Fisher’s exact test on the number of subjects. **d** Results from the microneutralization assays performed on the same donors shown in A). The neutralization capacity is expressed as the reciprocal of the highest dilution of the donor’ serum at which virus infection is blocked. **e** Gating strategy for the identification of plasmablasts (left panel) and paired analysis of the frequency of CD38^hi^CD27^hi^ plasmablasts in blood of young and healthy elderly individuals during the course of the response (D0-D28, right panel). Significant differences are expressed by * *p* < 0.05, ** *p* < 0.01, *** *p* < 0.001 and **** *p* < 0.0001

We vaccinated the healthy elderly and the young healthy volunteers with Vaxigrip© (Sanofi-Pasteur) following 2014/2015 seasonal recommendations and subsequently collected fasting blood specimens on day 0 (baseline, D0), day 2 (D2), day 7 (D7) and day 28 (D28). We then determined the hemagglutination-inhibition (HAI) antibody titer at baseline and D28 post-vaccination as shown in Fig. 1a. Both groups responded to vaccination with a significant increase in HAI titers against all three strains tested at day 28 (Fig. 1a). Following the current international guidelines for seroconversion (> 4-fold increase in HAI titers over baseline) and seroprotection (HAI titers ≥40) [19, 20], fewer young subjects had seroconverted compare with the elderly, which was primarily due to higher baseline titers in the young subjects (GMT values: Flu B: 411 ± 3 vs. 56 ± 4; H1N1: 149 ± 6 vs. 15 ± 5; and H3N2: 156 ± 4 vs. 38 ± 5 in young vs. elderly subjects). A high number of young individuals were already seroprotected at baseline against each of the three strains contained in the vaccine compared to the elderly (young versus old: 100% vs 63.6% against the B strain (*p* = 0.0005), 86.2% vs 40.9% against H3N2 (*p* = 0.001) and 72.4% vs 31.8% against H1N1 (*p* = 0.005)) (Fig. 1b). These high baseline titers may indicate previous exposure to influenza virus; subjects were excluded if they received an influenza vaccine within 6 months preceding the trial vaccination, however one cannot entirely rule out sustained protection from earlier vaccinations. A high baseline HAI titer has been reported to possibly interfere with TIV immunogenicity as measured by HAI titres [21] indicating that one should be cautious with interpreting the results of the seroconversion. Post-vaccination, the seroprotection for all three strains increased to 100% in the young and close to 100% in the healthy elderly, and there was no significant difference between the young and healthy elderly (Fig. 1c) in terms of seroprotection for any of the 3 strains (for H1N1: Fisher’s test *p* = 0.0739). Finally, when comparing the HAI titer endpoint at D28, older individuals display higher GMT for H3N2 (1159 vs. 610 with *p* = 0.0091), lower GMT for H1N1 (275 vs. 775 with *p* = 0.012) and no difference for FluB (1050 vs. 1726 with *p* > 0.05), which indicates no gross overall hyporesponsiveness to influenza vaccination in this group of selected healthy elderly subjects. These HAI titres results were corroborated by the microneutralization assays (Fig. 1d) showing that the influenza-specific antibodies produced following vaccination have a similar neutralization capacity in healthy elderly and young individuals. Based on these two different analyses (HAI and microneutralization assays), we can conclude that carefully selected healthy older adults are able to mount an antibody response quantitatively and qualitatively similar to young individuals.

## Plasmablast dynamic is sustained in healthy elderly

A major goal of this study was also to document the kinetics of cellular and humoral immune responses. Flow cytometry was used for immunophenotyping fresh whole blood to identify and monitor the recruitment of plasmablasts as well as assess their expansion and contraction phases from D0 to D28 (Fig. 1e). We reported the frequency of this populations as a ratio of the total immune cell fraction (CD45+). Firstly, in Fig. 1e we observed a significantly higher proportion of plasmablast cells (CD38^hi^CD27^hi^) in young subjects compared to elderly subjects at baseline (0.069% vs. 0.044%, *p* = 0.0063) that persists at D2 (*p* = 0.0013). This could be related to the higher baseline HAI titers in the young reported earlier (Fig. 1a), which suggests higher influenza virus/ vaccine exposure and/ or immunological memory. Thereafter, a significant increase in plasmablast frequencies from D2 to D7 (*p* < 0.001) was observed with a peak at D7, but no significant difference between the two age groups (0.226% vs 0.254%, *p* = 0.8690) was detected. However, the frequency of CD38^hi^CD27^hi^ plasmablasts increased more in the elderly (5.8 times) than in the young (3.3 times) from D0 to D7, mirroring the sharper HAI titer increase in the elderly. The frequency of plasmablasts diminished to baseline levels at D28 (0.078% in young and 0.061% in healthy elderly individuals). Overall, there is a clear cellular dynamic response observed after vaccination, which is preserved in healthy elderly Chinese Singaporeans.

## Conclusion

In our study, we demonstrate that overall, healthy elderly are fully capable to mount a robust humoral response via expansion of plasmablast, HAI response and neutralizing antibodies, comparable to that of young subjects. Herein, these data extends our previous findings [22] with community-living elderly subjects from Singapore, and demonstrates that humoral immunity after influenza vaccination is preserved in healthy elderly. The studied healthy elderly group showed all signs of aging as previously described, including a higher prevalence of persistent infections (eg. CMV), high levels of pro-inflammatory molecules (eg. CRP) and features of immune aging (higher frequencies of CD28^−^ and CD27^−^ T cells, data not shown). The fact that plasmablast population dynamics is preserved in the healthy elderly from the SLAS cohort confirms earlier studies utilizing the SENIEUR protocol for immunogerontological studies. An important question to address in the near future will involve identifying which age-associated co-morbidity may interfere with optimal vaccine responses in the elderly. We recently ruled out type-2 diabetes as a condition that may interfere with influenza vaccine responses in a study of community-living elderly in Canada [23]. Older adults commonly take statins to reduce cholesterol levels in order to manage cardiovascular risk, however recent published reports have raised concerns that statin use may impair vaccine-induced antibody responses, and reduce vaccine-induced protection, particularly for influenza A H3N2 [24]. Whether subjects with hypercholesterolemia or those on statin medication should be considered at-risk and benefit from improved vaccines is debatable, and in our study such subjects were excluded. Other conditions such as frailty have been proposed to alter influenza or pneumococcal vaccine responses, however this has also recently been challenged [25, 26]. Discrepancies in study outcome may be explained by different mean ages of the subjects included into the studies, the definition of frailty used, and living conditions (community vs. institution). A recent review highlighted the need to consider several approaches to stratify the older population in order to provide with the best vaccine strategy [27]. We are currently tackling these issues and other pertinent questions in an ongoing study aimed at understanding the associations between immunosenescence, hypo-responsiveness to vaccination and clinical phenotypes.

## Abbreviations

CMV: Cytomegalovirus
CRP: C-reactive protein
HAI: Hemagglutination-inhibition
HAI: Hemagglutination-inhibition
SLAS: Singapore Longitudinal Ageing Study
